# Alcohol- and Drug-Associated Injury Outcomes Among Older Adults Involved in Car Crashes

**DOI:** 10.1093/geroni/igab046.486

**Published:** 2021-12-17

**Authors:** Oluwaseun Adeyemi

**Affiliations:** University of North Carolina at Charlotte, Mankato, Minnesota, United States

## Abstract

Understanding how recent alcohol or drug use among older adults involved in car crashes can inform emergency care team on the morbidity and mortality risks. This study aimed to assess the odds of worsened health outcomes among older adults with evidence of alcohol or drug ingestion. This cross-sectional analysis used crash census data from the National EMS Information System. The outcome variable was the health outcome after EMS care, measured on a four-point ordinal scale: lower acuity, emergent, critical, and dead. The predictor variable was alcohol/drug use (present/not present). Age, race, gender, part of the body injured, and the revised trauma score of the patients were used as confounders. Odds ratio were calculated using proportional ordinal logistic regression. A total of 42,992 individuals, aged 65 years and older, were involved in car crash events, which required EMS activation. About 22.9% needed emergent care, 4.4% were classified as critical, and 0.4% died without resuscitation efforts. At the time of crash, 3.8% of the older population had evidence of alcohol or drug use. After adjusting for age, gender, race, injury location and revised trauma score of the crash patients, alcohol/drugs were associated with 54% increased odds of worse clinical outcome (AOR:1.54; 95% CI: 1.32-1.80). The adjusted odds remained elevated in urban (AOR: 1.69; 95% CI: 1.42-2.02) and suburban (AOR: 2.21; 95% CI: 1.12-4.35) and not significantly elevated in rural areas. Study findings can inform EMS service and emergency room care as well as policies that strengthen the urban and suburban EMS.

